# Burnout, anxiety and depression in secondary school teachers in Europe during the COVID-19 pandemic: a systematic scoping review & perspective of preventive occupational medicine

**DOI:** 10.1186/s12995-025-00488-z

**Published:** 2025-11-29

**Authors:** Ursula Wild, Sarah Herman, Marius König, Thomas C. Erren, Philip Lewis

**Affiliations:** Institute and Policlinic for Occupational Medicine, Environmental Medicine and Prevention Research, University Hospital of Cologne and University of Cologne, Cologne, Germany

**Keywords:** Teachers, COVID-19, Secondary school, Burnout, Anxiety, Depression

## Abstract

**Background:**

The COVID-19 pandemic added another layer of burden to what is already a demanding occupation; namely, secondary school teacher.

**Objective:**

To review the published literature concerning burnout, anxiety, and depression in secondary school teachers in Europe during the COVID-19 pandemic and to discuss the findings from the perspective of preventive occupational medicine.

**Methods:**

A systematic scoping review using the Medline and Web of Science databases with narrative synthesis of findings.

**Results:**

We identified 16 articles from seven European countries (Belgium, Bosnia and Herzegovina, Italy, Germany, Greece, Spain, Portugal) though results from one study were uninterpretable. Of the 15 remaining, seven assessed burnout or emotional exhaustion, seven assessed anxiety, and seven assessed depression (there was overlap). Only two studies were longitudinal and they focussed on burnout; the remaining 13 were cross-sectional in design. Questionnaires were used to assess severity scores. Mean severity scores for all outcomes appear to fluctuate across the pandemic, but are always high. Loss-to-follow up – indicated in the longitudinal studies – could mean a healthy worker effect (e.g. workers who fall ill and drop out of the study) biases both severity scores per se and their associations with other factors toward null in studies. Differences in- and associations with- outcome scores are reported for sex, age, job experience, cognitive-, personality-, and emotional intelligence-associated factors (e.g. extraversion, presenteeism, openness, resilience, clarity, repair), work-related factors (e.g. student issues, professional support, technology), and COVID-19 associated factors (e.g. family member vulnerability). There are differences in some findings across studies.

**Discussion:**

The COVID-19 pandemic presented a unique situation in which to study factors that contribute to -or provide protection against- burnout, anxiety, and depression. Female teachers may be a higher-risk group for depression. Potential factors that could be modified to mitigate outcomes, albeit identified as associating factors from mostly cross-sectional studies, include emotional intelligence training at the individual level and professional support at a systems level. Given the importance of the teaching profession and the high demands placed on teachers, future studies should consider interventions on such modifiable factors toward reducing health burden.

## Background

School teachers play an important role in society, educating and forming the future generation of adults. They also represent a large proportion of the general workforce (e.g., ~1.7% in Germany) [1, 2]. Yet, more teachers are needed in Europe, and it appears that this need shall not disappear soon [3]. According to a survey conducted among European teachers for the European Commission, 59% view the teaching profession as ‘unattractive’ or even ‘very unattractive’. Furthermore, 68% think that teacher workloads do not permit a healthy work-life balance [4]. Stress is identified as a major issue [4]. It is perhaps not surprising, that psychic and psychosomatic diseases are more common in the teacher’s profession than in other occupations [5]. Thus, school teachers can be identified as a large and important part of the workforce that require targeted support from occupational medicine.

The work experience for teachers during the COVID-19 pandemic was unique. A regular discussion topic in this period was whether schools should remain open for the benefit of education and to allow students to remain in their familiar surroundings or whether they should be temporarily closed to prevent spread of infection. Both on-site and off-site settings were challenging for teachers: on the one hand they were subjected to a higher risk of infection when working on-site whilst having some responsibility for the health of their classes. Depending on what restrictions were in a place at a given time-point and location, this could have included ensuring students were masked inside and outside, following ventilation guidelines such opening windows at regular intervals, administering SARS-CoV-2 rapid tests, checking their results, isolating positive students and calling parents/guardians for them to be picked-up from school). On the other hand, they had to provide lessons online at short notice when decisions were made to keep schools closed. Several studies from different countries have shown that the pandemic promoted psychiatric and psychological problems, like depression, anxiety, or substance abuse in the general population [6–8]. Following the pandemic, a 2023 survey in Germany finds that one third of the participant teachers reported regular emotional exhaustion (EE), and 10% reported daily EE [9]. Thus, there may be lessons that can be learned from the pandemic regarding factors that affect the occupational health of teachers.

From the background of school teachers at risk and the importance of this issue, the possibility to learn from the extremes during the pandemic, and the obligation to assess potential psychological hazards in the workplace, our aim was as follows: To examine the extent, range, and nature of the literature concerning burnout, anxiety, and depression in secondary school teachers in Europe during the COVID-19 pandemic by means of a systematic scoping review and to determine what can be of value for preventive occupational medicine. Given the abundance of literature considering all of primary, secondary and tertiary level teachers, we chose to focus on secondary school teachers in Europe to allow for a coherent and informative review of a particular occupational group.

## Methods

### Search strategy

We conducted a systematic scoping review of two literature databases (PubMed and Web of Science) to identify studies pertinent to our aim of examining the extent, range, and nature of the literature concerning burnout, depression, and anxiety in secondary school teachers in Europe during the COVID-19 pandemic years on 14^th^ May 2024. The review was not previously registered.

We informed our strategy with PICOS (Population, Intervention/Exposure, Comparator, Outcome, Study Design) definitions: (i) Secondary school teachers in Europe are the population of interest. (ii) There is no focus on a particular intervention or exposure for this project. The intention is to map any relevant interventions or exposures relevant to the study objective. (iii) Similar to intervention/exposure, no comparator is specified. (iv) The mental health outcomes-focus of this project concerns burnout, anxiety, and depression specifically. Regarding burnout, the focus is on burnout per se (i.e., as a whole) or EE; (i.e., as a major component of burnout). The other two components of burnout – namely, depersonalisation/cynicism and professional inefficacy were not a focus. The tools used to assess burnout place a greater focus on EE, EE has the best predictive power of burnout and is a more evident sign of burnout, it can be an easier dimension to address in terms of preventive occupational medicine. The other dimensions are also sometimes viewed as consequences of EE. (v) No specification was used concerning study design. All study designs were deemed potentially relevant.

### Screening

The search strings (Table 1) used to screen the databases were developed using terms pertinent to burnout, anxiety, and depression and long-COVID (which was identified as having similarities to the burnout syndrome at the time) and school teachers. The string was not limited by terms related to the COVID-19 pandemic or to Europe as a location. “Mental health” was not included as a search term due to being too non-specific. The returned studies metadata were downloaded in the Endnote^TM^ referencing software. Duplicates and studies not written in English or German were excluded. The titles and abstracts of remaining studies were then screened against pre-defined eligibility criteria (Table 1). Briefly, these include primary research studies of European secondary school teachers, the terms “Covid” OR “pandemic” mentioned in abstract and an indication of measured status/score/prevalence of burnout or anxiety or depression or measure of association with a potential predictor of the outcome. Excluded were articles at this stage which do not focus on secondary school teachers, studies of teachers of English as a foreign language only, studies outside Europe, publication pre-2020, and studies that validate assessment methods or well-being per se (without mention of burnout, anxiety, or depression) as topic. The full texts were then screened against further eligibility criteria (Table 1). Briefly, these include a European-based population, secondary school teachers, scores or categorisations of burnout (or EE as a symptom or major component of burnout), anxiety, or depression, and descriptive epidemiology or studies of association. Excluded – additional to the exclusion criteria for title & abstract – were studies comparing secondary school teachers to other teachers in other school levels without providing data on secondary school teachers specifically, studies of fear/anxiety regarding SARS-CoV-2 or COVID-19 infection/sickness per se, and studies that were conducted pre-COVID-19 pandemic. Disagreements in screening were discussed and resolved in the authorship group.

**Table 1:** Search string & eligibility criteria

PubMed & WoS	(depress* OR anxiety OR burnout OR “burn out” OR “mental exhaustion” OR “emotional exhaustion” OR “chronic fatigue” OR “long covid” OR “post covid” OR “chronic covid”) AND (teacher*[ti] OR educator*[ti] OR tutor*[ti] OR schoolteacher*[ti])
T&A Eligibility	*Inclusion Criteria* • Primary research studies, written in English of German, of European secondary school teachers. * “Covid” OR “pandemic” mentioned in abstract. • Indication of measured status/score/prevalence of depression or anxiety or burnout or measure of association with a potential predictor of the outcome. * *Exclusion Criteria* • Primary or University (Higher Education) level teachers. * • Studies of teachers of English as a foreign language only. • Non-European. * • Publication pre-2020. • Studies that validate assessment methods. • Well-being per se (without mention of depression, anxiety, or burnout) * If unclear whether the criteria (of Europe and/or secondary school teachers) were fulfilled, for instance if “teachers” per se was specified or if “worldwide” was specified, the study was included for full text analysis as it may contain stratified analyses relevant to the objective.
Full Text Eligibility	*Inclusion Criteria* • European-based population (and studied independently from non-European; e.g., if the study is worldwide, results for European-based strata must be available). • Secondary school teachers (and studied independently from non-secondary school teachers or educators from training centres; e.g., if the study concerns primary, secondary, and university level teachers, results for secondary level strata must be available). The secondary school can be private, comprehensive, or special needs. ** • Scores or categorisations of depression, anxiety, or burnout (or “exhaustion”). • Descriptive epidemiology or studies of association. These can include measures of prevalence or incidence or comparisons thereof (e.g., prevalence pre-pandemic vs pandemic vs post-pandemic) or measures of association *Exclusion Criteria* • Studies of teachers of English as a foreign language only. • Studies comparing secondary school teachers to other teachers in other school levels without providing data on secondary school teachers specifically. • Studies of fear/anxiety regarding SARS-CoV-2 or COVID-19 infection/sickness per se. • Studies of well-being per se that do not mention anxiety, depression, or burnout Studies that were conducted pre-COVID-19 pandemic per se. ** It should be noted that “secondary school teachers” may not always be explicitly stated in this form. Thus, also included are teachers of students from 10+ years or from 5^th^ grade until before university if student ages or grade levels are indicated.

T&A = Title and abstract, WoS = Web of Science Core Collection

### Data extraction & synthesis

Data extraction included study identifying information, population and location data, study design, analyses conducted and methods used, and results. P-values < 0.05 were considered as statistically significant (independent of the threshold values used by the authors of the respective studies). The extent, range, and nature of the literature concerning burnout, depression, and anxiety in secondary school teachers in Europe during the COVID-19 pandemic years is then presented in a narrative synthesis. Perspective on what might be of value for occupational medicine is presented in the discussion. Discrepancies in data extraction were discussed and resolved in the authorship group.

## Results

### Brief overview

**Fig. 1:**
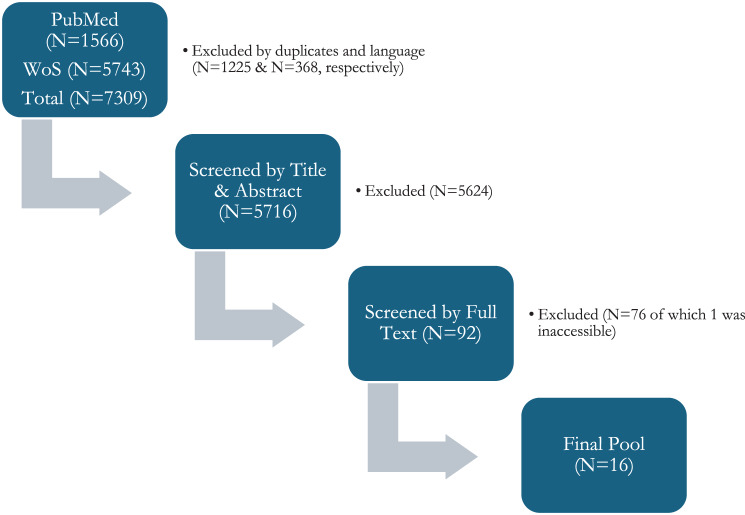
Prisma flow diagram. WOS = web of science

The screening of the databases yielded 7,309 articles. After screening, a final pool of 16 articles remained for synthesis. A Prisma Flow diagram depicting the flow of articles through the screening stages is presented in Fig. 1.

Following narrative critical appraisal, one study was excluded because results about secondary school teachers are not interpretable [10]; thus, 15 studies are included in this synthesis. Of these, seven articles addressed burnout, two of which used a longitudinal design (from Germany [11] and Belgium [12]. The other five cross-sectional studies were conducted in Spain [13–15], Germany [16], and Portugal [17]. Regarding anxiety, the seven cross-sectional studies were conducted in Spain [18, 19], Italy [20–22], Germany [16], and Bosnia and Herzegovina [23]. For depression, the seven cross-sectional studies were conducted in Spain [19, 24], Italy [20, 22], Germany [16], Bosnia and Herzegovina [23], and Greece [25]. Some studies considered multiple outcomes; thus, there is some overlap. There were no studies that focussed on COVID-19-related anxiety alone (i.e., all studies that included COVID-19-related anxiety also included other factors associated with anxiety and were thus eligible for inclusion in the current review).

The following synthesis is divided into sections concerning burnout, anxiety, and depression, respectively. As such, some studies are synthesised in multiple sections. Overviews of the studies are presented in Tables 2 , 3, 4 on burnout, anxiety, and depression, respectively.

**Table 2:** Overview of included studies regarding burnout

Author (Year)	Location	Population*	Study Details	Analyses	Results
**Longitudinal**					
Voss (2023) [11]	Germany	*N* = 856 secondary school: pre-service for time points 1–2, in service from time point 3 Female = 65.76% Age = 27.9 (4.17) years	MBI (2007–2022)	Severity, pre vs during pandemic, sex, student difficulties, personality, support, technical equipment	↑EE during pandemic. ↑EE with difficulties with students, teacher’s extraversion. ↓EE with better technical equipment at school and teacher’s openness. ↔EE with support from principal, collaboration with colleagues, support from parents, neuroticism, or sex.
De Laet (2022) [12]	Flanders, Belgium	*N* = 2,167 secondary school Female = 77.6% Age = 42 (10.2) years	MBI (adapted) (Sep 19 – Aug 21)	Severity, Change over time (vs previous time point).	High risk of burnout syndrome in autumn 2019 in 20.8% of sample. Highest risk of burnout syndrome at beginning of summer 2021 = 30.8%. Burnout and EE risk follow similar patterns: autumn 2019, ↑winter 2019, ↓first lockdown 2020, ↑beginning of summer 2020, ↓summer holidays 2020, ↑spring 2021, ↑beginning of summer 2021, ↓summer holidays 2021.
**Cross-Sectional**					
Martínez-Ramon (2023) [13]	South-east Spain	*N* = 401 secondary school Female = 70.6% Age = 44.36 (9.38) years	MBI (Jan-March 2021)	Severity, sex	↑EE in females [mean 3.2 (SD 1.6)] compared to males [2.71 (SD 1.6)].
Koestner (2022) [16]	Germany	*N* = 31,089 (15,137 or 43.5% secondary school) Female = 77.5% Age = 45.8 (10.5) years	MBI (March 2021)	Severity, Pre vs during pandemic.	Mean EE = 2.53–2.62 (SD 1.74–1.76) [different for secondary school types]. ↑EE from pre to during pandemic: 3.69–3.82 (SD 0.89–0.94). ^†^
Da Costa Ferreira (2021) [17]	Portugal	*N* = 1,044 grades 5–12 Female = 76.6% Age = 51.05 (7.35) years	CBI (2nd school trimester, 2020)	Severity, sex, age, cyberbullying, presenteeism.	Mean burnout 3.08 (SD 0.8). ↑Burnout in females. ↔Burnout with age. ↑Burnout with cyberbullying (mild correlation) and productivity loss due to presenteeism (mild-moderate correlation). Productivity loss due to presenteeism may mediate the association cyberbullying and burnout.
Martínez-Ramon (2021) [14]	Murcia, Spain	*N* = 419 secondary school Female = 69.7% Age = 44.37 (9.39) years	MBI (Jan/Feb 2021)	Severity, resilience, COVID-19	Burnout prevalence = 30.8%. ↓Burnout with resilience (mild correlation). ↑EE with COVID-19 factors.
Sánchez-Pujalte (2021) [15]	Spain	*N* = 430 high school Female = 46.28% Age = 41.4 (11.07) years	MBI (“worked during pandemic in 2020/2021”)	Severity, sex, age, experience, emotional intelligence.	High level of EE in 60.2% of sample, medium level in 11.9%, low level in 27.9%. ↔EE by sex. ↓EE with age and job experience. ↓EE with clarity, repair, regulation, and empathy dimensions of emotional intelligence (mild correlation). ↔EE with attention or emotional autonomy (correlation).

*Age is given as mean (standard deviation) unless otherwise specified. †We are unsure if there is a typo in the scale they used and the question asked was: How would you describe this aspect in comparison to before the COVID-19 pandemic? CBI = Copenhagen Burnout Inventory; EE = Emotional Exhaustion; MBI = Maslach Burnout Inventory

**Table 3:** Overview of included studies regarding anxiety (all cross-sectional)

Author (Year)	Location	Population*	Study Details	Analyses	Results
Garvey (2023) [18]	Spain	*N* = 196 secondary school Female = 74.1% Age = unknown	GAD-7 (Apr/May 2020)	Severity.	“Normal” anxiety in < 25% of participants.
Levante (2023) [20]	Italy	*N* = 242 middle school Female = 87% Age = 46.2 (10.4) years	DASS-21 (Sep 21-Jan 22)	Severity, sex, age, experience, emotional processing, job satisfaction.	Mean female 1.39 (SD 0.4), male 1.23 (SD 0.27). ↓Anxiety with emotion processing ability and job satisfaction (mild correlation). ↔Anxiety with age or teaching experience (correlation).
Koestner (2022) [16]	Germany	*N* = 16,184 secondary school Female = 77.5% Age = 45.8 (10.5) years	GAD-2 (March 2021)	Severity	Mean 1.94–2.04 (SD 1.64–1.68).
Cadamuro (2021) [21]	Reggio Emilia, Northern Italy	*N* = 83 high school Female = 75.9% Mean age = 53.47 years	STAI (unknown)	Severtiy, age, sex, meta-cognitive experiences, ICT beliefs.	Mean 2.49 (SD 0.53). ↔Anxiety association with age, sex, metacognitive experiences, or ICT beliefs.
Kovac (2021) [23]	Bosnia and Herzegovina	*N* = 151 high school Female = 84.3% Age = 20+ years	DASS-21 (October 2021)	Severity.	“Normal” anxiety reported by 57.6%, “elevated” levels by 42.4%.
Ozamiz-Etxebarria (2021) [19]	Basque Autonomous Community and Navarre, Spain	*N* = 491secondary school Female = 79.7% Age = 42.6 (9.96) years	DASS-21 (Sep 2020)	Severity.	Mean 3.95 (SD 3.5).
Truzoli (2021) [22]	Lombardy, Italy	*N* = 107 high school Female = 64.5% Age = 49.8 (10.1) years	BAI (Apr/May 2020)	Severity, sex, locus of control behaviour.	Mean 11.9 (SD 10.1), ~50% scoring moderate-severe. ↔Anxiety association with sex. ↑Anxiety with locus of control behaviour (moderate correlation).

Age is given as mean (standard deviation) unless otherwise specified; BAI = Beck Anxiety Inventory; DASS-21 = Depression, Anxiety and Stress Scale including 21 items; GAD = Generalized Anxiety Disorder; ICT = Information and Communication technologies; STAI = State-Trait Anxiety Scale

**Table 4:** Overview of included studies regarding depression (all cross-sectional)

Author (Year)	Location	Population*	Study Design	Analyses	Results
Levante (2023) [20]	Italy	*N* = 466 (242 or 54.3% middle school) Female = 87% Age = 46.2 (10.4) years	DASS-21 (Sep 21-Jan 22)	Severity, age, experience, emotion processing, job satisfaction	Mean 1.52 (SD 0.55), female mean 1.54 (SD 0.55), male mean 1.46 (SD 0.53). ↓Depression with emotion processing ability (mild-moderate correlation), job satisfaction (mild-moderate correlation), age (mild correlation). ↔Depression with teaching experience (correlation).
Sánchez-Pujalte (2023) [24]	Madrid, Spain	*N* = 430 secondary school Female = 46.28% Age = 41.4 (11.07) years	PHQ-9 (“worked during pandemic in 2020/2021”)	Severity, sex, age, experience, emotional intelligence.	“Moderate/severe” depression in 16.2% of sample. Mean female 1.96 (SD 1.04), male = 1.62 (SD 0.88). ↑Depression association with sex. ↓Depression associated with clarity and repair. ↔Depression associated with age and experience. Unclear association with attention.
Koestner (2022) [16]	Germany	*N* = 31,089 (15,137 or 43.5% secondary school) Female = 77.5% Age = 45.8 (10.5) years	PHQ-2 (March 2021)	Severity.	Mean 1.92–1.95 (SD 1.42–1.44).
Kovac (2021) [23]	Bosnia and Herzegovina	*N* = 559 (151 or 27% high school) Female = 84.3%	DASS-21 (October 2021)	Severity.	“Elevated” Depression in 35.8% of participants, “Normal” in 64.2%.
Ozamiz-Etxebarria (2021) [19]	Basque Autonomous Community and Navarre, Spain	*N* = 1633 (491 or 30.1% secondary school) Female = 79.7% Age = 42.6 (9.96) years	DASS-21 (Sep 2020)	Severity.	Mean 3.81 (SD 4.01).
Truzoli (2021) [22]	Lombardy, Italy	*N* = 107 high school Female = 64.5% Age = 49.8 (10.1) years	CES-D (Apr/May 2020)	Severity, sex, external locus of behaviour, online teaching satisfaction, COVID-19.	Mean 16.3 (SD 9.5). ↔Depression association with sex. ↑Depression with external locus of behaviour (moderate correlation). ↓Depression with online teaching satisfaction (mild). ↔Depression with family member affected by COVID-19 (correlation).
Stachteas (2020) [25]	Volos, Greece	*N* = 266 high school Female = 63% Age = 89% < 60 years	One question (March 2020)	Severity, sex, age, education level, subject specialty, COVID-19.	Severity category proportions skewed towards “not at all” and “rarely depressed”. ↑Depression with female (proportion χ^2^ analysis). ↔Depression with age, subject specialty, education level, being member of a vulnerable group, family member belonging to a vulnerable group, or living with a minor in the family (proportion χ^2^ analysis).

Age is given as mean (standard deviation) unless otherwise specified; CES-D = Center for Epidemiological Studies Depression Scale; DASS-21 = Depression, Anxiety and Stress Scale; EE = Emotional Exhaustion; PHQ = Patient Health Questionnaire

### Synthesis

#### Burnout

An overview of the studies concerning burnout is presented in Table 2. All studies used the Maslach Burnout Inventory (MBI; 16–22 items pertaining to different dimensions of burnout [incl. EE], with seven levels of frequency ratings from never to a few times per year [least frequent] to daily [most frequent]) or a shortened version of the MBI to assess burnout or EE, except the study by Ferreira et al. (2021) from Portugal who used an adapted version of the Copenhagen Burnout Inventory (CBI; 19 items rated on 5-point Likert scales from “never/almost never” to “always”). Study population sizes were between 401 and 31,089 in total, but were often smaller at different time points of measurement because of loss to follow up in longitudinal studies or because of stratification into different school levels (the number of participants included for each statistical test is not always clear). All but one study had more than 50% female participants. Mean burnout or EE severity scores or counts of categorized severity scores (e.g. higher vs. lower scores) are explored in all seven studies, severity score changes over time are explored in three studies, sex differences in three studies, age and job experiences in two studies, teacher cognitive/emotional-related factors in four studies, student-related issues in two studies, technical equipment and professional- and social- support both in one study, and COVID-19 associated factors in one study. These are synthesised in the following paragraphs.

In terms of severity scores over time, De Laet et al. (2022) in Belgium included the most time points [12]. They used categorisations of scores to indicate risk of burnout and EE. Risks increased from the start of term 2019 into winter 2019–2020, dropped shortly after the first lock down (i.e., the move to online teaching), increased across May-June 2020, then dropped in the 2020 summer holidays. At the next follow-up in early 2021, risks were higher than in 2020, and reached their highest levels in May-June 2021 before dropping for the 2021 summer holidays. Of note, the loss to follow-up at each time point was extensive, indicating that a “healthy-worker effect” may need to be considered when drawing conclusions from all studies conducted during the pandemic. In the other longitudinal study by Voss et al. (2023) in Germany, EE increased from summer 2019 to summer 2020 (most participation in the surveys occurred before summer holidays and when schools were partly closed) and remained higher into Spring 2022 after school closures had ended but many restrictions remained in place [11]. The change in mean severity of the outcome across the study population can be considered as increasing from low-moderate to moderate-high. The cross-sectional study by Koestner et al. (2022) in Germany also included a temporal component insofar as EE difference was assessed from the question “How would you describe this aspect in comparison to before the COVID-19 pandemic?” [16]. The reported result indicates less often than before the pandemic, which is different to the two longitudinal studies, but this is not discussed in their text and it is unclear why this might be so. Nonetheless, the severity score for March 2021 per se could be categorised as moderate.

The three cross-sectional studies from Spain also report severe or high levels of EE. Two studies from Martinez-Ramon et al. (2021, 2023) report scores indicating moderate to severe EE in January-March 2021 [13, 14]; however, it is not clear if the population they analysed in their 2023 publication included some or all of the population from their 2021 publication. The study by Sanchez-Pujalte et al. (2021) uses categorized scores for those who “worked during the pandemic in 2020/2021” and identified ~60% in their high level of EE category [13, 17]. The last cross-sectional study from Portugal by Da Costa Ferreira et al. (2021) report data from the second trimester 2020 (presumably, this means the school trimester that begins in January); however, it is unclear how to interpret the reported score of 3.08 [17]. Despite some limitations, the severity scores appear to change over time with the highest mean scores identified in first half of the year in 2021, suggesting the risk of burnout became more extreme as the pandemic progressed.

Two cross-sectional studies identified higher EE scores in females, while one study identified no difference [15]. One longitudinal study identified no effect-modification by sex on changes in severity of EE over time (immediately pre- vs. during the pandemic) [11]. Lower EE scores were associated with older age and more years of experience in one study [15], but not with age in another [17].

Cognitive-, personality-, and emotional-related factors explored in four studies. Extraversion was associated with a steeper increase in EE from pre to during the pandemic, while more openness (i.e., having more personal emotional resources) was associated with a less pronounced increase, and neuroticism appeared to have no impact in one study [11]. One study reports burnout correlated mildly with productivity loss due to presenteeism [17], another reports a mild inverse correlation with resilience [14], and another reports a moderate inverse correlation with clarity and repair, regulation, and empathy [15]. No correlations were observed between EE and attention, pro-sociality, and emotional autonomy.

Two studies report higher levels of EE/burnout related to difficulties with students [11, 21]; the latter specifies the difficulty being observing cyberbullying in students [17]. One study reports that better technical equipment at schools is associated with smaller changes in EE from pre- to during pandemic periods but support from leadership, colleagues, and parents did not play a role [11]. Regarding COVID-19 associated factors, one study reports increased presence of EE with increased COVID-19 experiences (e.g., affecting family or friends) [14].

#### Anxiety

An overview of the seven cross-sectional studies concerning anxiety is presented in Table 3. Three used the DASS-21 (Depression, Anxiety and Stress Scale; seven of 21 items assess anxiety and another seven items assess depression over the past week, with ratings based on 4-point Likert scales from “did not apply” to “applied very much”) to assess anxiety [19, 20, 23]; two studies used the GAD-7 (General Anxiety Disorder, containing seven items to rate on a 4-point Likert scale from “not at all” to “nearly every day”) [18] and the GAD-2 (two items about feeling nervous/anxious and not being able to stop worrying) [16] questionnaires, one study used the BAI (Beck Anxiety Inventory; 21 items and, similar to above, with scores from 0 to 3 for each) [22], and one study used the STAI-S (State-Trait-Anxiety Inventory Scale; 20 items and uses 4-point Likert scales from “almost never” to “almost always”) and a question about how teachers feel when thinking about remote teaching [21]. Study population sizes and sex distribution was similar to that of burnout. Outcome severities are explored in all seven studies, sex differences in three, and age and job experience in two. Three studies consider cognitive-, personality-, and emotional-related factors and one study considers COVID-19-associated factors.

In terms of DASS-21 severity scores, Levante et al. (2023) report a mean item score of 1.35 (severe) for September 2021 to January 2022 in Italy [20], Ozamiz-Etxebarria et al. (2021) report a mean score of 3.95 (either “normal” or unclear severity meaning) for September 2020 in Spain [19], and Kovac et al. (2021) – using categories only – report “elevated” anxiety levels in 42.4% and “normal anxiety” in 57.6% for October 2021 in Bosnia and Herzegovina [23]. Garvey et al. (2023) report normal anxiety levels in < 25% April-May 2020 in Spain using the GAD-7 [18]. Koestner et al. (2022) report “medium” levels of 2.53–2.62 for March 2021 in Germany using the GAD-2 and describe this as exceeding the anxiety level of the general population (though the values supporting this statement in the abstract and main text do not match) [16]. From Italy but with data collection period unknown, Cadamuro et al. (2021) report a mean score of 2.49 (indicating moderate anxiety) using the STAI-S [21]. Also from Italy for April-May 2020, Truzoli et al. (2021) report a mean score of 11.9 (indicating mild, but 25% of participants can be categorized as moderate to severe anxiety) using the BAI [22]. While the anxiety scores are high across studies, we cannot infer about fluctuations over the course of the pandemic due to the different tools used.

Two studies did not identify sex differences [21, 22], but one study reports higher mean anxiety in females although this is not assessed statistically and we cannot assess this ourselves as observation counts are not provided alongside the means and standard deviations [20]. Two studies find no association with age or years of experience [20, 21]. Overall, it is unclear whether there are sex or age associations with anxiety.

One study reports mild inverse correlations of anxiety with processing emotions and job satisfaction [20], another observes no association with teachers’ beliefs about ICT or metacognitive experiences [21], and another reports a moderate correlation between anxiety and locus of control behaviour [22]. One study presents high percentages of participants reporting more negative perceptions of being personally and professionally affected by COVID-19 lockdowns and relatively larger percentages of these reporting moderate and severe anxiety, but without formal statistical analyses [22].

Regarding Cadamuro et al. (2021), their text cites their Table 7 as relevant but this is not correct [21]; presumably the Table in their “Table 8 position” is intended although this is labelled as a second Table 3 and with “Students” in the Table header but “Teachers” in the Table legend. This Table data matches what is described in the text, so we use this in our synthesis above.

#### Depression

An overview of the seven cross-sectional studies concerning depression is presented in Table 4. Three of the seven studies used the DASS-21 [19, 20, 23]. Two studies used the PHQ-9 (Patient Health Questionnaire; 9 items and uses 4-point Likert scales to rate items “not at all” to “nearly every day”) [24] and the PHQ-2 (uses two items) [16], respectively. One study used the CES-D (Center for Epidemiologic Studies Depression Scale; 20 items and uses 4-point Likert scales from “never” to “mostly”) [22], and another used a single question about depression and a 6-point Likert scale (“not at all” to “almost always”) [25]. Study population sizes were similar to burnout, but female participants exceeded 60 or even 70%. Outcome severity is explored in all seven studies. Sex, age/job experience, cognitive-, personality-, and emotional-related factors, and work-related factors are considered in three studies each. Two studies consider COVID-19-associated factors.

Regarding DASS-21 severity scores, Ozamiz-Etxebarria et al. (2021) report a mean value of 3.81 (either “normal” or unclear severity meaning) from September 2020 in Spain [19], whereas Levante et al. (2023) report a mean score of 1.52 (moderate-extreme) from September 2021 to January 2022 (the “2^nd^ wave of the pandemic”) in Italy [20]. Kovac et al. report “elevated” levels in 35.8% and “normal” levels in 64.2% from October 2021 in Bosnia & Herzegovina using the DASS-21, but did not report the cut-offs used [23]. Using the PHQ-9, Sánchez-Pujalte et al. (2023) report 16.2% of their secondary school teachers can be categorised as presenting with at least “moderate” depression from 2020/2021 in Spain (24). Koestner et al. (2021) report a mean score of 1.93 for March in Germany using the PHQ-2 [16], stating the scores exceeded the level of the general population (means of 1.93 vs. 1.24 for females, 1.72 vs. 1.03 for males), but these might be considered mild. Using the CES-D, Truzoli et al. (2021) report a mean score of 16.3 (mild severity) with about 40% of the population indicating possible depression from April-May 2020 in Italy [22]. Stachteas et al. (2020) from Greece report that ~75% indicated less than “a little” depression while ~25% indicated at least having “sometimes” symptoms of depression using their single question in March 2020 [25].

Regarding sex, one study reports a higher mean score for females than for males, but this is not tested statistically and we cannot compare means as the numbers of observations are not provided [20]. Two studies found statistically worse depression scores for females compared to males [24, 25], but one study did not [22]. One study reports a negative correlation with age, but no correlation with teaching experience [20], and two studies report no associations with age or years of experience [24, 25].

One study reports depression symptoms inversely correlated with emotional processing ability [20], another reports correlation with locus of control [22]. One study observed inverse correlations with clarity and repair but not attention, inverse associations with all three when included in a regression model, but a change in direction for the association with attention when burnout components are added to the regression model. We cannot reach a conclusion for attention, but inverse associations with clarity and repair held [24].

Regarding work-related factors, job satisfaction and online teaching satisfaction were inversely correlated with depression in one study each [20, 22]. Another study reports no association with teaching specialty or educational level [25]. Regarding COVID-19-associated factors, one study found no correlation with living with a minor, being personally highly vulnerable, or having a highly vulnerable family member [25]. Another study observed no correlation with having a family member affected by COVID-19 [22].

#### Associations between burnout/emotional exhaustion, anxiety, and depression

Two studies report correlation between anxiety and depression [20, 22]. One study reports an association between EE and depression [24]. Some studies consider other components of burnout but they are beyond the scope of this review.

## Discussion

In summary, the most alarming finding is the prevalence of high severity scores for burnout in secondary school teachers in Europe during the COVID-19 pandemic. A possible healthy worker effect (in this case, whereby workers who fall ill during the pandemic drop out of the study) effect may imply that these high severity scores are in fact underestimations. Possible areas of intervention in terms of preventive occupational medicine are suggested (“suggested” meaning factors are associated/correlated with outcomes) at both individual and systems levels. These include technical equipment and support, emotional intelligence and locus of control training, and added focus on vulnerable groups (female).

### The COVID-19 Pandemic

There is evidence of increasing severity of mental health issues as the COVID-19 pandemic progressed. This is clearer for burnout and EE, which have been assessed in longitudinal studies. When considering the data collection periods chronologically, this may also be the interpretation for anxiety. Studies that collected their data in the early phase of the pandemic and before the widespread social restrictions or “lockdowns” in 2020 present mean anxiety scores that could be considered normal or mild [18, 19, 22], while more severe scores are identified for 2021 and 2022 [16, 20]. This is less clear when considering the studies that use categorisations with < 25% report “normal” anxiety in 2020 [18], but ~50% categorised as normal in 2021 [23]. Of course, the different tools used and pandemic burden at the particular times in the particular locations make it difficult to get a clearer picture. Depression appears more unchanged with normal/mild severities reported during 2020 and 2021 and similar proportions of “normal” categorizations [16, 19, 22, 23]. According to the WHO the COVID-19 pandemic triggered a 25% increase in the prevalence of anxiety and depression globally [26]; thus, this finding might be considered unexpected. One study reports more severe scores for September 2021 to January 2022, but season may also play a role in depression severity scores then [20]. Of course, for all three outcomes, severity scores could be increasingly biased towards the null as the pandemic progresses because of a healthy worker effect; thus, if depression were expected as a more severe manifestation of an external stressor, this finding of no change may not be unexpected.

A recent meta-analysis from Ozamiz-Etxebarria et al. (2023) analysed prevalences of burnout among teachers during the pandemic from cross-sectional studies [27]. They found a pooled prevalence of 52% (95% CI 33–71%) and they state that this value is higher than what was found for health professionals. The findings from Ozamiz-Etxebarria et al. (2023) not only add support to our rationale (albeit including primary and tertiary level educators, too), but they are in line with higher severity scores observed across the pandemic in this review, which adds a validity to the findings regarding the factors tested for associations with the outcomes.

### Factors associated with outcomes

The most commonly study factors for associations with the outcomes are non-modifiable characteristics such as sex, age, and years of work experience. In line with estimated whole population prevalences with higher percentage rates for women for depression (6% female vs. 4% male) [28], female teachers appear at higher risk for higher severity scores compared to males [24, 25]. Sex differences in burnout and anxiety severity scores are less clear with studies indicating higher scores in females [13, 17, 20], and others no difference [11, 15, 21, 22]. Higher scores in females might be expected given population prevalences for anxiety (33% female vs. 22% male [29]) and – at least in some European countries – for burnout [30]. There are no clear differences by age or work experience, which is perhaps unexpected. On the one hand, younger teachers may be able to better cope with digital demands on working online but older teachers who are still in their jobs may be more resilient (healthy worker effect). There may be other confounding variables.

Potentially modifiable characteristics associated with outcomes include emotional intelligence at the individual level [11, 20, 22, 24] while digitalisation aspects are potentially modifiable systems level characteristics [11, 17]. One study reporting productivity loss due to presenteeism as a mediator of the association between student cyberbullying and teacher burnout suggests further links in conceivable chains of causation [17] (with cyberbullying linked to digitalisation; digitalisation being the incorporation of new digital technologies to transform work – important for e.g., teaching online).

### Limitations

Regarding the review per se, two key points warrant discussion; namely, (i) focus on secondary school teachers only, and (ii) focus on burnout and EE specifically. For (i), it is important to note that only studies providing stratified data for secondary school teachers were included and only stratified data were extracted and synthesised. Many excluded studies and data from included studies considered different teacher levels (i.e., primary and secondary levels) together; thus, some information on factors potentially associated with outcomes may have been missed. However, our justification for this strategy and what can be considered a strength is that focusing on this specific group may allow clearer comparisons of severity of outcomes and factors associated with severity given, for instance, more comparable work conditions. As examples of differences, primary school teachers typically focus on one class of pupils across the whole day (indeed, for the year), whereas secondary school teachers are more specialised in their subjects and may teach different classes of students. Classroom management will also differ by age groups. With regard to anti-infection measures, such disparities in work conditions could be important. For (ii), a limitation can be that burnout was only assessed using the search words “burnout” or “emotional exhaustion”, the latter being one of the three dimensions of burnout (along with depersonalisation/cynicism and professional efficacy). Some studies may have been missed. However, pilot searches (e.g., using [(depersonalisation AND teacher AND covid) NOT (burnout)] as a search string, which yielded *n* = 0 articles on PubMed) suggest missing relevant studies is likely to result in few, if any. Furthermore, although the other dimensions add different aspects to burnout, they are not used on their own for assessment of burnout whereas emotional exhaustion sometimes is. Moreover, that few to no studies were found in the screening process that used only one of these two dimensions to assess burnout also supports the focus of the review on burnout and emotional exhaustion not being a major limitation.

We note that not including “mental health” as a search term may result in some studies that include burnout, anxiety, and depression slipping through the net; however, as “mental health” is a catch-all term, we accept the trade-off between precision and sensitivity.

Regarding the studies’ findings and understanding the clinical impact on teachers it is important to note that the assessment tool scores do not equate to clinical diagnoses and comparing severity scores and proportions of participants in different severity categories in different places and at different times cannot be done cleanly. The healthy worker effect has also been described above.

Lastly, regarding the studies’ findings and providing perspectives for preventive occupational medicine (below), the identified factors of interest for preventive occupational medicine were often analysed as simple correlations and from cross-sectional studies. As per what we posit above regarding lack of associations between age/years of experience and outcome severities (technology capabilities, older healthy worker resilience), unaccounted for effect modification (e.g., age) of the associations between modifiable characteristics and severity scores may be an issue.

### Perspectives for preventive occupational medicine

Akin to more extreme environments or more extreme experimental interventions used for measurable effects in basic research, situations like the COVID-19 pandemic represent extremes for workers that should be explored. Such situations represent unique natural experiments [31]. We hypothesized/envisaged that increased burnout, anxiety, and depression severity could make factors associated with mitigating their severity more identifiable. In addition, we targeted higher-risk groups of secondary school teachers for comparability.

Towards targeted support from preventive occupational medicine, this review identifies female teachers whose needs and circumstance may differ, especially regarding burnout [24, 25]. In terms of interventions on modifiable factors, the findings from this review point to supporting digitalisation in terms of not only equipment [11], but also skills (using technology), culture (which includes for instance, sending and receiving messages outside of typical school day hours), and strategy (how to use and lead others to use technology) [17]. Training in terms of emotional processing abilities may also be warranted [11, 15, 20, 22, 24].

Interventive research could include assessing how digitalisation-related aspects and emotional processing abilities and interventions thereon may be affected by, for instance, teacher age. A further worthwhile and low-cost option for research could be to revisit existing studies that considered different levels of education together and conduct stratified analyses now. One such study is the German study by Koestner et al. (2022), which has a notably large sample size (which seems difficult to achieve given the sample sizes presented in other studies) yet provides only minimal descriptive data pertinent to the focus of this review [16]. Revisiting such studies may be a worthwhile endeavour to avoid research waste.

Possibilities for research that were not identified in this literature include, for instance, that teachers had added responsibilities toward the health of their students in the context of a poorly understood pandemic, or that many female teachers may have added burden as primary carers in their respective families.

Lastly, it is worth following up on what factors that changed from pre-pandemic to during-pandemic are still relevant and may be causing burden post-pandemic, such as aspects of digitalisation.

## Conclusion

Secondary school teachers have an important and demanding job. Extreme conditions that can cause (or exacerbate outcomes to facilitate) measurable responses must be thoroughly researched. Burnout has been particularly problematic for secondary school teachers in Europe during the pandemic. Targets identified for preventive occupational medicine include improved digitalisation- and emotional intelligence-related aspects.

## Data Availability

Data sharing is not applicable to this article as no datasets were generated or analysed during the current study.

## Abbreviations

BAI: Beck Anxiety Inventory
DASS-21: Depression, Anxiety and Stress Scale including 21 items
CBI: Copenhagen Burnout Inventory
CES-D: Center for Epidemiological Studies Depression Scale
DASS-21: Depression, Anxiety and Stress Scale including 21 items
EE: Emotional Exhaustion
GAD: Generalized Anxiety Disorder
GLM: Generalized Linear Model
ICT: Information and Communication technologies
LG(C)M: Latent Growth (Curve) Model
MBI: Maslach Burnout Inventory
PHQ: Patient Health Questionnaire
STAI: State Anxiety Scale
WoS: Web of Science
